# 643. Dynamic ^11^C-PABA PET/CT Imaging for Visualizing Pulmonary *Mycobacteroides abscessus* Infection: Animal and Human studies

**DOI:** 10.1093/ofid/ofae631.208

**Published:** 2025-01-29

**Authors:** Yuderleys Masias-Leon, Oscar J Nino-Meza, Amy Kronenberg, Kelly Flavahan, Carlos E Ruiz-Gonzalez, Elizabeth Tucker, Nikki Parrish, Gyanu Lamichhane, Noah Lechtzin, Sanjay K Jain

**Affiliations:** Johns Hopkins University School of Medicine, Baltimore, Maryland, United States., Baltimore, MD; Johns Hopkins University School of Medicine, Baltimore, Maryland; Johns Hopkins, Baltimore, Maryland; Johns Hopkins, Baltimore, Maryland; Johns Hopkins University/School of Medicine, Glen Burnie, Maryland; Johns Hopkins, Baltimore, Maryland; Johns Hopkins University/School of Medicine, Glen Burnie, Maryland; Johns Hopkins University/School of Medicine, Glen Burnie, Maryland; Johns Hopkins University/School of Medicine, Glen Burnie, Maryland; Johns Hopkins Children's Center, Baltimore, MD

## Abstract

**Background:**

Non-tuberculous mycobacteria (NTM) primarily affect patients with structural lung damage with *Mycobacteroides abscessus* being the second most prevalent NTM in the U.S. Conventional imaging tools cannot distinguish infection from underlying inflammation, and definitive diagnosis often requires invasive procedures. Therefore, development of noninvasive bacteria-specific diagnostics that differentiate a true infection from inflammation, often present in these patients, would be highly useful clinically.

3H-PABA accumulation in bacteria.

**Figure:**
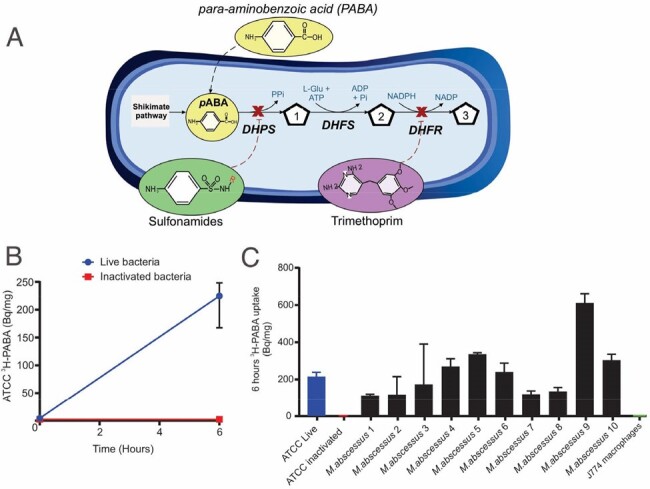

A. Folate biosynthesis pathway in bacteria is absent in mammalian cells, providing a target for antibiotics (sulfonamides and trimethoprim) as well as for developing novel metabolism-derived bacteria-specific imaging approaches. B. In vitro uptake of 3H-PABA by M. abscessus ATCC 19977 strain. There is no accumulation in heat-inactivated bacteria or mammalian cells. C. In vitro uptake of 3H-PABA (6 hours of incubation) in ten random M. abscessus clinical isolates (some with antibacterial resistance) from the Johns Hopkins Hospitals demonstrates robust uptake in all strains suggesting that this is a conserved process and is not affected by antibiotic resistance. Six technical replicates from each culture were used for these experiments. Data is shown as median and interquartile range (IQR) in Becquerel (Bq) per mg of protein. PABA, para-aminobenzoic acid. DHPS, dihydropteroate synthase. DHFS, dihydrofolate synthase. DHFR, dihydrofolate reductase. 1, dihydropteroate. 2, dihydrofolate. 3, tetrahydrofolate. ATCC, American Type Culture Collection.

**Methods:**

*In vitro* uptake assays were performed to test the incorporation of ^3^H-ParaAminoBenzoicAcid (PABA), a bacteria-specific tracer develop by our laboratory, in *M. abscessus* ATCC strain and ten random clinical isolates (Johns Hopkins Hospitals). Dynamic ^11^C-PABA positron emission tomography (PET) was performed in a mouse model of *M. abscessus* pulmonary infection and in a patient with microbiologically-confirmed *M. abscessus* pulmonary infection (NCT05611905).

Dynamic 11C-PABA PET/CT in a mouse model of pulmonary M. abscessus infection.

**Figure:**
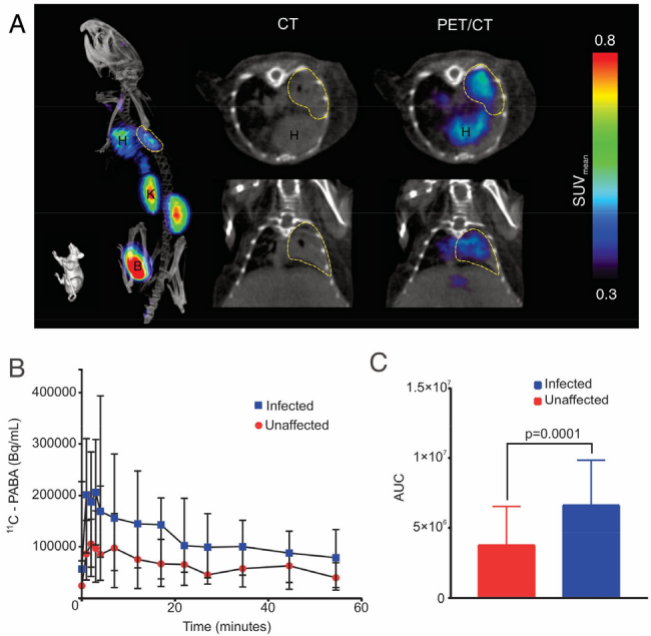

A. Representative images from a mouse at 4 weeks after an aerosol infection with M. abscessus. Whole-body PET/CT (left) shows the tracer distribution, demonstrating PET signal from the kidneys and the bladder due to renal excretion of 11C-PABA (chemically identical to PABA). The pulmonary infection is highlighted in yellow and the 11C-PABA PET signal colocalizes with the M. abscessus infection noted on the CT. B. Time activity curve (TAC) demonstrating higher 11C-PABA accumulation in the infected compared to unaffected lung regions over the full 60 min (n = 7 mice). The sustained 11C-PABA PET uptake in the dynamic studies is highly suggestive of specific uptake, given that PET signals arising from nonspecific capillary leak (noted in areas of inflammation and infection) would dissipate over time, as the tracer is eliminated from the circulation. C. Area under the curve (AUC) over the 60 min shows significantly higher PET uptake in the infected lesion (P < 0.01; Wilcoxon non-parametric analysis, n = 7 mice). Data is shown as median and interquartile range (IQR) in Becquerel (Bq) per milliliter (mL). CT, computed tomography. PET, positron emission tomography. H, heart. K, kidney. B, bladder. SUV, standardized uptake values. IQR, interquartile range.

**Results:**

All bacterial strains demonstrated rapid and substantial accumulation of ^3^H-PABA (**Fig. 1**). Dynamic ^11^C-PABA PET demonstrated sustained accumulation in lung lesions versus the unaffected lung tissues over the 60 min scan (*P* < 0.01; n = 7 mice) (**Fig. 2**). First-in-human dynamic ^11^C-PABA PET in a 33-year-old female with cystic fibrosis and microbiologically confirmed *M. abscessus* pulmonary infection performed per the U.S. FDA guidance, demonstrates significantly higher PET uptake in the infected lesion (*P* = 0.02; n = 5 lesions) (**Fig. 3**). The sustained ^11^C-PABA PET uptake in the animal and human dynamic studies is suggestive of specific uptake, given that PET signals arising from nonspecific capillary leak (noted in areas of inflammation and infection) would dissipate over time, as the tracer is eliminated from the circulation.

Dynamic 11C-PABA PET/CT in a patient with M. abscessus pulmonary infection.

**Figure:**
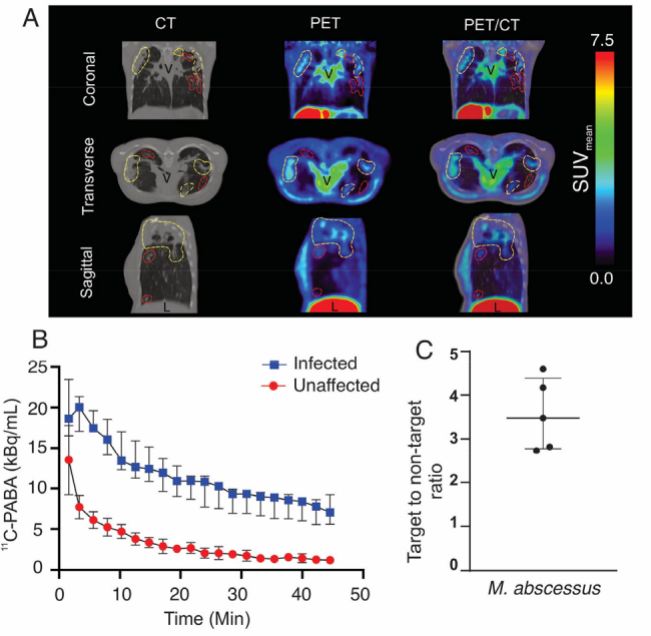

Dynamic 11C-PABA PET/CT in a 33-year-old female with cystic fibrosis and microbiologically confirmed M. abscessus pulmonary infection performed in accordance with U.S. FDA guidelines from an ongoing study approved by the Johns Hopkins Hospitals IRB (clincialtrials.gov NCT05611905). A. 11C-PABA PET/CT images are shown. 11C-PABA PET signal colocalizes with the lesions noted on the CT (outlined in yellow) but not all lesions visualized on CT are PET avid (outlined in red), suggesting that some lesions may be sterile or have much lower bacterial burdens. B. TAC demonstrating higher 11C-PABA accumulation in the infected compared to unaffected lung regions. The sustained 11C-PABA PET uptake in the dynamic studies is highly suggestive of specific uptake, given that PET signals arising from nonspecific capillary leak (noted in areas of inflammation and infection) would dissipate over time, as the tracer is eliminated from the circulation. C. AUC target (affected lung area) to non-target (unaffected lung area) demonstrates significantly higher PET uptake in the infected lesion (P = 0.02; Wilcoxon non-parametric analysis, n = 5 lesions). CT, computed tomography. PET, positron emission tomography. L, Liver. V, vessels. SUV, standardized uptake values. IQR, interquartile range. Bq, Becquerel.

**Conclusion:**

^11^C-PABA PET is an innovative, noninvasive bacteria-specific diagnostic tool with clinical potential to differentiate *M. abscessus* infections from inflammation in patients with structural lung damage and underlying disease. This tool could also be helpful in monitoring treatment responses and for enabling precision medicine and individualized care for patients with complicated infections.

**Disclosures:**

**Sanjay K. Jain, MD**, Fujirebio Diagnostics, Inc. (Malvern PA): Grant/Research Support|Novobiotics, LLC: Advisor/Consultant|Novobiotics, LLC: Grant/Research Support|T3 Pharmaceuticals (Basel, Switzerland): Grant/Research Support

